# Evaluating the effect of setup uncertainty reduction and adaptation to geometric changes on normal tissue complication probability using online adaptive head and neck intensity modulated proton therapy

**DOI:** 10.1088/1361-6560/acd433

**Published:** 2023-05-30

**Authors:** Arthur Lalonde, Mislav Bobić, Gregory C Sharp, Ibrahim Chamseddine, Brian Winey, Harald Paganetti

**Affiliations:** 1 Department of Radiation Oncology, Massachusetts General Hospital and Harvard Medical School, Boston, Massachusetts, United States of America; 2 ETH Zürich, Zürich, Switzerland

**Keywords:** adaptive proton therapy, normal tissue complication probability, image guided proton therapy, head and neck cancers, cone-beam CT

## Abstract

*Objective*. To evaluate the impact of setup uncertainty reduction (SUR) and adaptation to geometrical changes (AGC) on normal tissue complication probability (NTCP) when using online adaptive head and neck intensity modulated proton therapy (IMPT). *Approach.* A cohort of ten retrospective head and neck cancer patients with daily scatter corrected cone-beam CT (CBCT) was studied. For each patient, two IMPT treatment plans were created: one with a 3 mm setup uncertainty robustness setting and one with no explicit setup robustness. Both plans were recalculated on the daily CBCT considering three scenarios: the robust plan without adaptation, the non-robust plan without adaptation and the non-robust plan with daily online adaptation. Online-adaptation was simulated using an in-house developed workflow based on GPU-accelerated Monte Carlo dose calculation and partial spot-intensity re-optimization. Dose distributions associated with each scenario were accumulated on the planning CT, where NTCP models for six toxicities were applied. NTCP values from each scenario were intercompared to quantify the reduction in toxicity risk induced by SUR alone, AGC alone and SUR and AGC combined. Finally, a decision tree was implemented to assess the clinical significance of the toxicity reduction associated with each mechanism. *Main results*. For most patients, clinically meaningful NTCP reductions were only achieved when SUR and AGC were performed together. In these conditions, total reductions in NTCP of up to 30.48 pp were obtained, with noticeable NTCP reductions for aspiration, dysphagia and xerostomia (mean reductions of 8.25, 5.42 and 5.12 pp respectively). While SUR had a generally larger impact than AGC on NTCP reductions, SUR alone did not induce clinically meaningful toxicity reductions in any patient, compared to only one for AGC alone. *Significance* Online adaptive head and neck proton therapy can only yield clinically significant reductions in the risk of long-term side effects when combining the benefits of SUR and AGC.

## Introduction

Intensity modulated proton therapy (IMPT) has the potential to reduce the risk of radiation-induced side effects associated with conventional photon radiotherapy while achieving equivalent or superior tumor control (Blanchard *et al* 2016a, Leeman *et al* 2017, Moreno *et al* 2019). This is especially relevant for the treatment of head and neck cancers, where common side effects such as xerostomia and dysphagia can significantly impair quality of life (Langendijk *et al* 2008). To identify the patients that would benefit the most from the toxicity reductions associated with proton therapy, model-based approaches using normal tissue complication probability (NTCP) have been suggested (Langendijk *et al* 2013, Widder *et al* 2016). A recent implementation of a model-based patient selection system suggested that 35% of all head and neck patients eligible for initial treatment plan comparison would qualify for proton therapy due to an expected clinically significant reduction in NTCP (Tambas *et al* 2020).

NTCP reductions associated with proton therapy are obtained by leveraging the sharp dose falloff created at the distal edge of the Bragg peak. However, this feature also makes proton therapy more sensitive than photon radiotherapy to inter-fractional geometrical changes such as random non-rigid anatomical variations (e.g. jaw positioning, head rotation, neck folds, etc) and treatment-related changes (e.g. weight loss, tumor shrinkage, etc) (Müller *et al* 2015, Stützer *et al* 2017). While a loss in target coverage is usually the main concern associated with geometrical changes, they can also lead to an over-dosage of the organs at risk (OARs) surrounding the target volume. This was highlighted by Stützer *et al* (2017) who reported, after 20 fractions, an increase in the median dose to the ipsilateral parotid gland of 3.4 Gy for proton therapy, compared to 0.8 Gy for photon radiotherapy. Likewise, (Müller *et al* 2015) reported a reduction in the conformity index of the 95% isodose of up to 15% for proton therapy plans compared to less than 5% for photon radiotherapy in head and neck patients after 10 fractions.

The usual approach to mitigate the impact of inter-fractional setup variations and anatomical changes on IMPT dose distributions is to increase the robustness of the treatment plan upfront, using robust optimization (Van De Water *et al* 2016, Cubillos-Mesías *et al* 2019). However, gains in plan robustness are often achieved at the cost of an increase in NTCP (Van De Water *et al* 2016), potentially compromising the benefits of IMPT. As an alternative, online adaptive proton therapy workflows have been proposed by different groups (Da Silva *et al* 2015, Jagt *et al* 2017, Botas *et al* 2018, Matter *et al* 2019, Lalonde *et al* 2021, Paganetti *et al* 2021, Bobić *et al* 2021, 2023). Online adaptation (OA) corrects for anatomical changes and setup variations by adapting the treatment plan just before delivering each fraction, using either a fast re-optimization (Botas *et al* 2018, Matter *et al* 2019, Lalonde *et al* 2021, Paganetti *et al* 2021, Bobić *et al* 2021, 2023) or dose restoration (Da Silva *et al* 2015, Jagt *et al* 2017, Bernatowicz *et al* 2018) technique. The potential benefits of online adaptive proton therapy in terms of NTCP mitigation are twofold: first, it might reduce the need for large setup uncertainty margins (Jagt *et al* 2017, Lalonde *et al* 2021) and second, it has the potential to account for geometrical changes that would result in an increased dose to OARs. When both effects are leveraged, online adaptive proton therapy has been shown to yield a lower dose to most OARs while maintaining superior target coverage than state-of-the-art robust optimization methods (Lalonde *et al* 2021). The potential impact of online adaptive proton therapy on NTCP has however not been quantified yet.

The aim of this work was to assess and quantify the reductions in toxicity risks achievable using online adaptive head and neck IMPT, as well as the individual contributions of setup uncertainty reduction (SUR) and adaptation to geometrical changes (AGC) on the total NTCP reductions. For this purpose, a retrospective cohort of ten head and neck squamous cell carcinoma patients with daily cone-beam CT (CBCT) was used to compare NTCP for six toxicities using three distinct scenarios. Differences in NTCP between each scenario was evaluated to assess the impact of SUR alone, AGC alone and SUR and AGC combined. Finally, the clinical significance of NTCP reductions associated with each mechanism was established using a decision tree inspired by a model-based patient selection algorithm.

## Methods and materials

### Patient data

Our patient cohort consisted of ten head and neck squamous cell carcinoma patients treated at the Massachusetts General Hospital with volumetric modulated arc therapy, since CBCT imaging was not available for our proton therapy patients at the time of this study. Each patient dataset consisted of a planning CT acquired on a wide bore GE scanner (General Electric Medical Systems, Milwaukee, WI) as well as a series of daily CBCT obtained on an Elekta XVI system (Elekta AB, Stockholm, Sweden) using a 100 kVp tube voltage and a 220-degree acquisition. The number of CBCTs available for each patient ranged between 30 and 35, for a total of 328 scans analyzed. No patient of this cohort had their treatment interrupted and no offline replanning was deemed necessary during the treatment courses.

Two clinical target volumes (CTV) were delineated on each planning CT by a trained radiation oncologist: a high-risk CTV including the primary tumor and high-risk lymph nodes, as well as a low-risk CTV, including bilateral lymph nodes considered at risk for subclinical disease. The constrictor muscles, larynx, oral cavity, spinal cord, brainstem, esophagus and both parotid glands were delineated on the planning CT. The esophagus, constrictor muscles and larynx were contoured following published guidelines for swallowing OARs (Christianen *et al* 2011), while internal protocols were used to define the other OARs.

### Treatment planning

Two different treatment plans were created for each patient in RayStation (v8.99, Raysearch Laboratories, Stockholm, Sweden). The first plan was robustly optimized to both CTVs using the minimax method (Fredriksson *et al* 2011) and considering a 3 mm isotropic setup uncertainty. The second plan was also optimized to both CTVs, but without using explicit setup robustness constraint. As done in our previous studies (Lalonde *et al* 2021, Bobić *et al* 2021, 2023), other sources of uncertainty not directly addressed by online adaption (e.g. range uncertainty, variable RBE, delineation/registration accuracy) were assumed to be accounted for by the original planning technique. Treatment planning objectives were defined similarly as in previous *in silico* studies on IMPT for head and neck squamous cell carcinoma patients (Cubillos-Mesías *et al* 2019, Lalonde *et al* 2021). Plans were optimized using a simultaneously integrated boost, with a prescribed dose of 57 and 70 Gy to the low and high risk CTVs respectively. The treatment plans were optimized to meet the following dose constraints to both target volumes: *D*_98%_ ≥ 95% and *D*_2%_ ≤ 107% of the prescription dose. For the OARs, the following dose constraints were used: *D*_mean_ < 26 Gy to each parotid gland, *D*_mean_ < 42 Gy to the constrictor muscle, *D*_mean_ < 40 Gy to the larynx, *D*_max_ < 45 Gy to the spinal cord and *D*_max_ < 54 Gy to the brainstem.

All plans were optimized using multi-criteria optimization considering all OARs listed above as well as the esophagus and the oral cavity, for which the dose was minimized without compromising target coverage. Treatment planning was done using the *IBA Dedicated Nozzle* beam model with beam angles of 60°, 180° and 300°, a 40 mm range shifter, 30 mm minimum air gap and a spot spacing factor of 1. The Monte Carlo algorithm of RayStation was used for plan dose calculation using a 2.0 × 2.0 × 2.0 mm^3^ dose grid and a relative biological effectiveness of 1.1.

### Dose accumulation and OA

**Figure 1.**
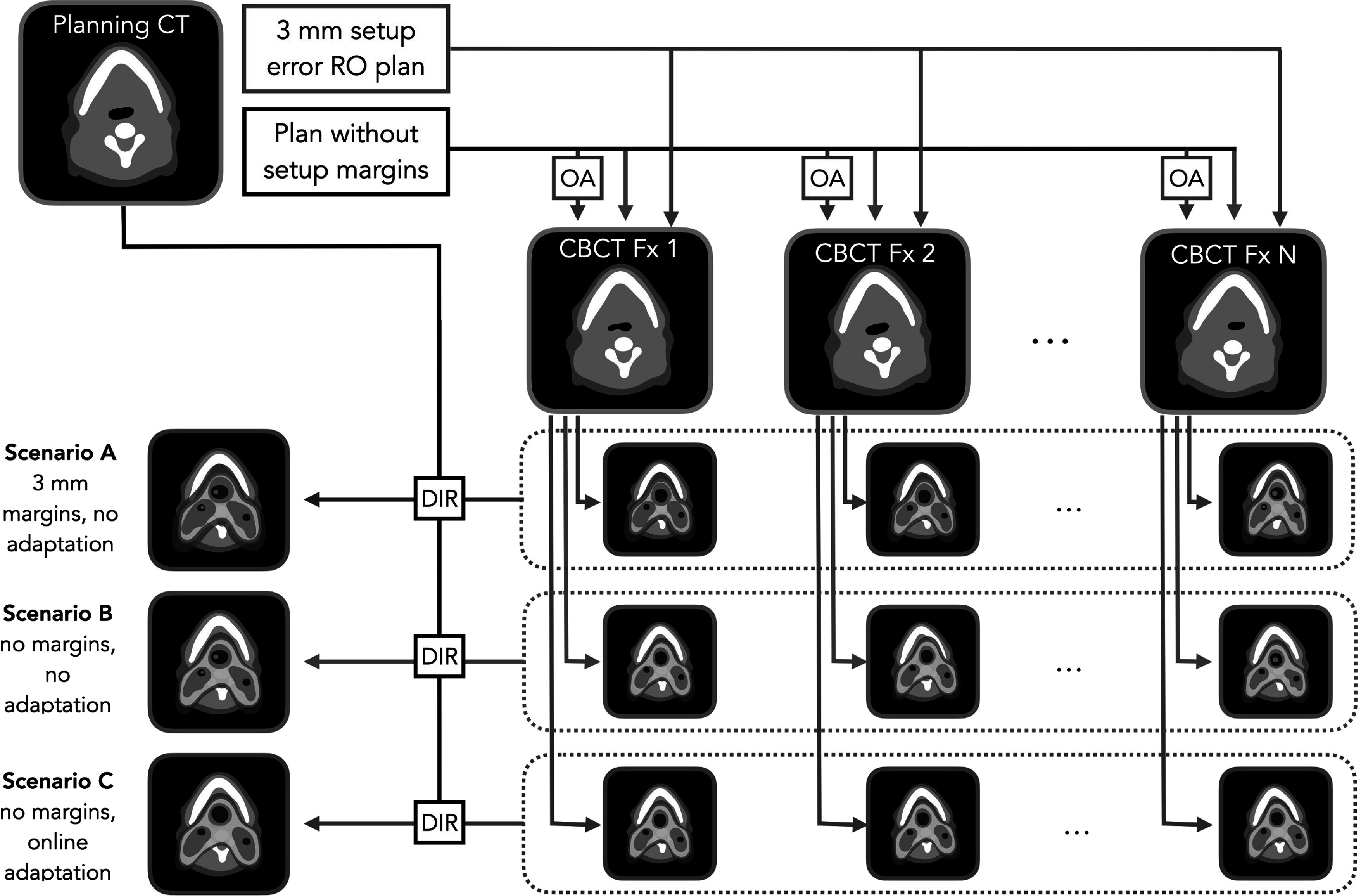
Schematic representation of the different scenarios compared in this work. Abbreviations: CBCT = cone-beam CT, OA = online adaptation, RO = robust optimization, DIR = deformable image registration, Fx = fraction.

The three scenarios illustrated in figure 1 were considered in this work. Scenario A consisted of the robust plan used at each fraction without adaptation, scenario B the non-robust plan used without adaptation and scenario C, the non-robust plan used with daily OA. As detailed in the following section, patient-specific results associated with each scenario were intercompared rather than analyzed individually, this to assess the specific impact on NTCP of SUR alone, AGC alone as well as SUR and AGC combined.

For scenario C, OA of the treatment plans was done using a constrained spot-intensity re-optimization approach developed within our group and detailed in previous publications (Lalonde *et al* 2021, Bobić *et al* 2021, 2023). This method restores the plan quality by only re-optimizing the weights of a subset of highly weighted proton beamlets, without modifying their position or energy. This makes OA fast and potentially avoids elaborate quality assurance procedures compared to full re-optimization, but limits the possibility to fully restore the initial plan quality in cases of drastic anatomical changes. The beamlets selected for optimization were defined as the smallest subset of beamlets carrying at least 33% of the total spot weights, with the constraint that the number of beamlets selected represented at least 10% of the total number of spots. The same constraints and objectives as for the initial treatment planning were used to re-optimize the plans.

Plan adaptation was based on the daily scatter corrected CBCT images. Scatter correction was performed in the projection domain using a Monte Carlo trained deep convolutional neural network previously validated for head and neck proton therapy (Lalonde *et al* 2020). Once corrected for scatter, the CBCT images were rigidly registered (using 6 degrees of freedom) to the planning CT focussing on a region of interest encompassing the high-risk CTV and the spine, mimicking our in-room clinical setup. From there, a set of daily contours were generated using deformable image registration (DIR) between the planning CT and the scatter corrected CBCTs in Plastimatch (Sharp *et al* 2010). Visual inspection of the propagated contours was done before each adaptation. Re-optimization of the selected beamlets’ weight was done using Opt-4D (Trofimov *et al* 2005), while dose calculation was performed using gPMC (Qin *et al* 2016), a GPU accelerated dose calculation algorithm, using the same beam model as in RayStation. The median time for adaptation including DIR and dose calculation was 12 min, ranging from 8 to 22 min within our patient cohort. For all patients, dose distributions associated with each scenario were calculated on the daily scatter-corrected CBCTs and accumulated on the planning CT using DIR. Dose calculation on the daily CBCTs was performed using RayStation (with Monte Carlo) for scenario B, while gPMC was used for scenarios A and C. This way, any potential discrepancies between gPMC and RayStation would be intrinsically addressed by AGC without affecting scenario B.

### NTCP calculation and evaluation

NTCP models for acute oral mucositis of grade ≥ 3 (Bhide *et al* 2012), xerostomia lasting 12 months after therapy (Houweling *et al* 2010), patient reported swallowing dysfunctions for solids and liquids (Christianen *et al* 2012), physician-rated dysphagia of grade ≥ 2 (Christianen *et al* 2012) and aspiration assessed by video-fluoroscopy (Eisbruch *et al* 2011) were applied to the cumulative dose distributions associated with each scenario. NTCP models and parameters used for each toxicity are summarized in table 1, derived from reference (Jakobi *et al* 2015). From the three scenario-specific NTCP values, toxicity reductions associated with SUR, AGC and SUR and AGC performed together were calculated as follows for each patient:

\begin{eqnarray*}\begin{array}{l}{\mathrm{\Delta }}NTC{P}_{SUR}=NTC{P}_{B}-NTC{P}_{A}\\ {\mathrm{\Delta }}NTC{P}_{AGC}=NTC{P}_{C}-NTC{P}_{B}\\ {\mathrm{\Delta }}NTC{P}_{SUR\,\&amp;\,AGC}=NTC{P}_{C}-NTC{P}_{A}\end{array}\end{eqnarray*}

where ${\mathrm{NTCP}}_{X}$ represents the NTCP associated with the cumulative dose distribution from scenario *X* as detailed in figure 1 and ${\mathrm{\Delta NTCP}}_{Y}\,$refers to the NTCP reduction induced by mechanism *Y*. Of note, while ${\mathrm{\Delta NTCP}}_{\mathrm{AGC}}$ could in theory be calculated from any realistic baseline setup robustness (e.g. 3 mm instead of 0), we decided to limit our analysis to the three scenarios described above for the sake of clarity and simplicity. The same applies to ${\mathrm{\Delta NTCP}}_{\mathrm{SUR}},$ which could also have been evaluated from a different setup uncertainty pair (e.g. going from 4 to 1 mm). Previous work has however demonstrated that NTCP for common head and neck toxicities was linearly related to setup uncertainty settings between 1 and 5 mm (Wagenaar *et al* 2021), indicating that our evaluation should be representative of any SUR of 3 mm within that range. To evaluate whether the linearity of NTCP with the setup uncertainty setting was indeed preserved down to 0 mm, an additional scenario considering a 1 mm setup uncertainty setting and no OA was considered and compared to scenarios A and B. Results for this analysis are presented in the appendix.

**Table 1.** NTCP models implemented in this work.

Toxicity	NTCP model	Parameter values
Oral mucositis	$\mathrm{NTCP}={\left(1+{\left(\frac{{D}_{50}}{D}\right)}^{k}\right)}^{-1}$	*D* = *D*_mean_ of oral mucosa
		*D*_50_ = 51 Gy
		*k* = 1
Xerostomia	$\mathrm{NTCP}={\mathrm{\Phi }}\left(\frac{g\mathrm{EUD}\left(n\right)-{D}_{50}}{m\,\cdot {D}_{50}}\right)$	*n* = 1 for contralateral parotid gland dose
		*D*_50_ = 39.9 Gy
		*m* = 0.4
Swallowing dysfunction—solids	$\mathrm{NTCP}={\left(1+{e}^{a-b\cdot {X}_{1}-c\cdot {X}_{2}}\right)}^{-1}$	*X*_1_ = *D*_mean_ of supraglottic larynx
		*X*_2_ = 1
		*a* = 5.98
		*b* = 0.074 Gy^−1^
		*c* = −1.209
Swallowing dysfunction—liquids	$\mathrm{NTCP}={\left(1+{e}^{a-b\cdot {X}_{1}-c\cdot {X}_{2}}\right)}^{-1}$	*X*_1_ = *D*_mean_ of superior PCM
		*X*_2_ = *D*_mean_ of supraglottic larynx
		*a* = 6.89
		*b* = 0.049 Gy^−1^
		*c* = 0.048 Gy^−1^
Dysphagia	$\mathrm{NTCP}={\left(1+{e}^{a-b\cdot {X}_{1}-c\cdot {X}_{2}}\right)}^{-1}$	*X*_1_ = *D*_mean_ of superior PCM
		*X*_2_ = *D*_mean_ of supraglottic larynx
		*a* = 6.09
		*b* = 0.057 Gy^−1^
		*c* = 0.037 Gy^−1^
Aspiration	$\mathrm{NTCP}={\mathrm{\Phi }}\left(\frac{g\mathrm{EUD}\left(n\right)-{D}_{50}}{m\,\cdot \,{D}_{50}}\right)$	*n* = 1 for larynx dose
		*D*_50_ = 46.5 Gy
		*m* = 0.5

**Figure 2.**
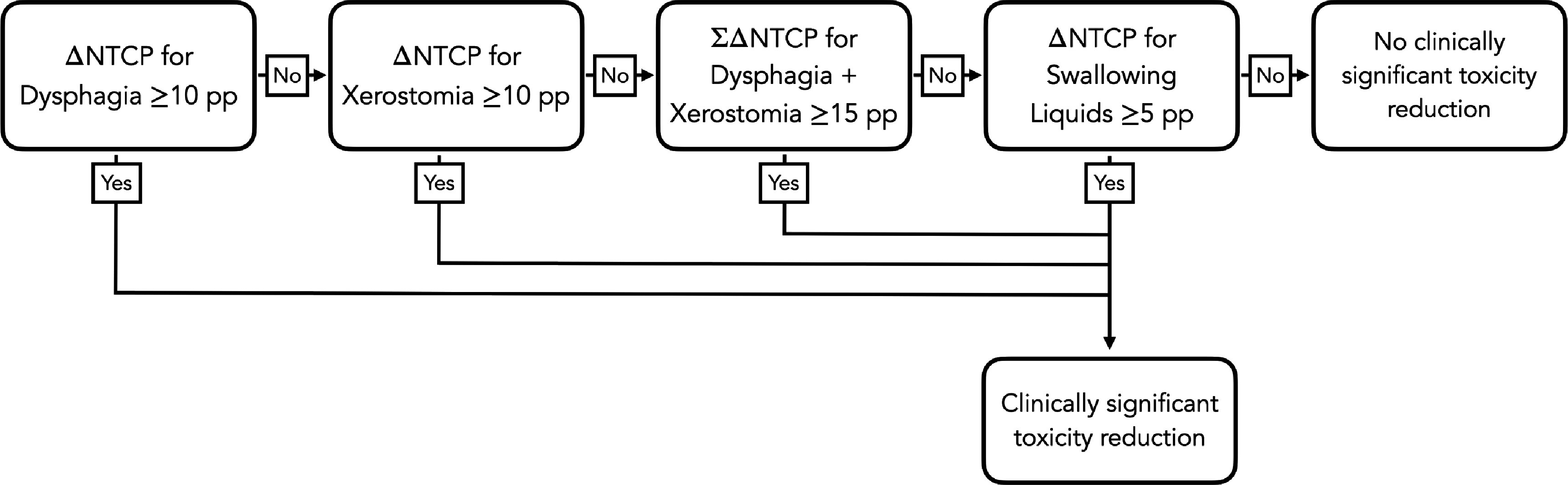
Decision tree used to establish the clinical significance of the toxicity reductions induced by each mechanism studied in this work.

Finally, the clinical significance of NTCP reductions associated with each mechanism was established using a decision tree illustrated and figure 2 and inspired by the one used in the Dutch model-based selection system (Tambas *et al* 2020). Although in this work no form of patient triage was performed, the decision tree was used to quantify the number of patients for whom a given mechanism induced a clinically meaningful NTCP reduction.

## Results

**Figure 3.**
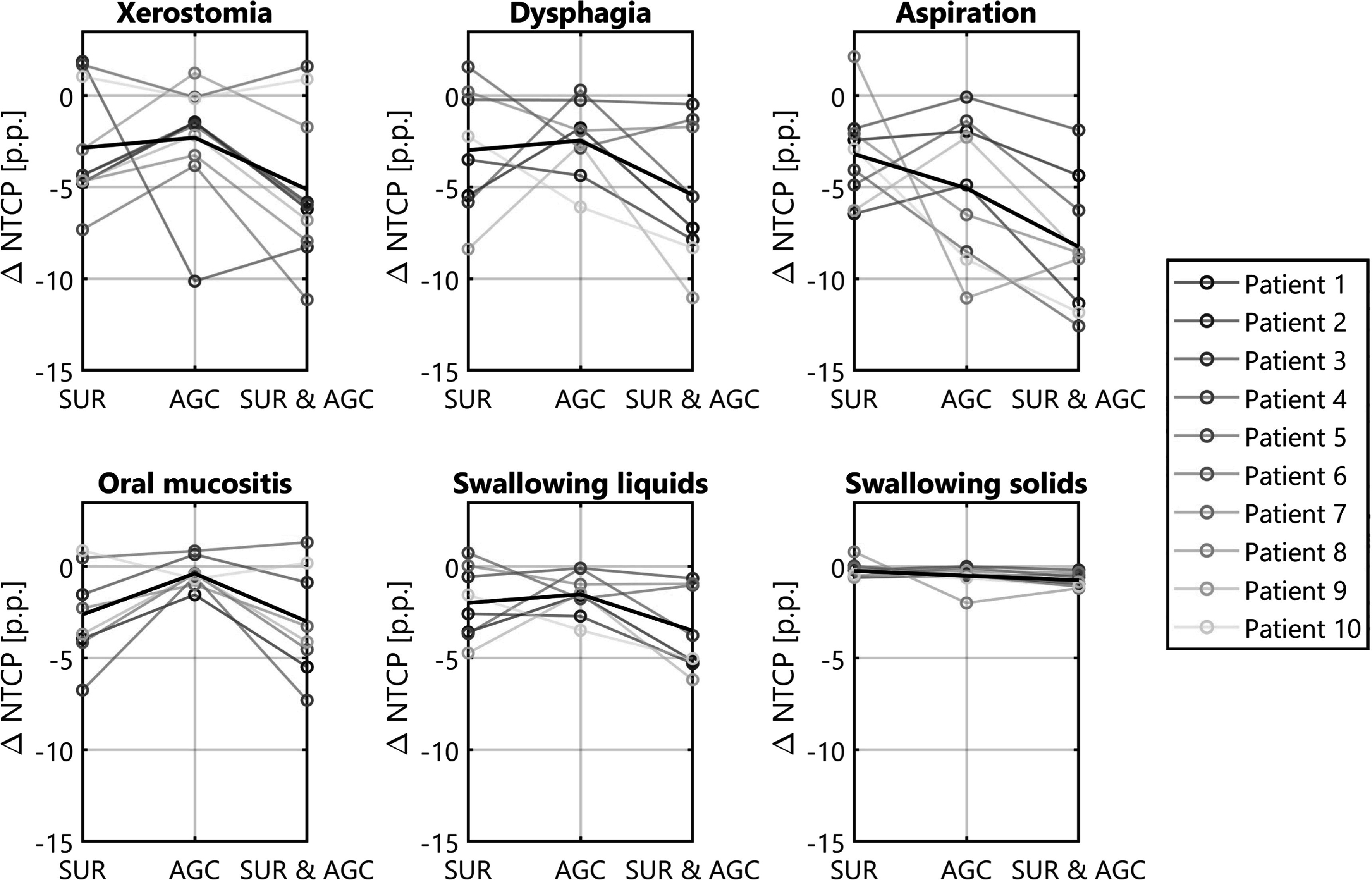
Normal tissue complication probability (NTCP) differences induced by a 3 mm setup uncertainty reduction (SUR) and adaptation to geometrical changes (AGC) as well as SUR and AGC combined. Each line represents one patient, while the black lines report the mean of the cohort.

NTCP reductions associated with SUR, AGC and SUR and AGC combined, as calculated with equation (1), are presented for each toxicity in figure 3. Each colored line represents one patient while the thick black line reports the mean value of the cohort. Missing lines correspond to patients for whom an OAR needed for NTCP calculation was fully encompassed by one of the target volumes. With very few exceptions, NTCP differences in figure 3 are negative, indicating that both SUR and AGC have the potential to reduce the risk for all toxicities considered in this work. Note that ΔNTCP can be positive in cases where the reduced setup robustness makes the plan more vulnerable to anatomical changes or when OA has to compromise OAR sparing in order to maintain tumor coverage. With the exception of aspiration, results also indicate that NTCP reductions achievable with SUR are generally larger than with AGC alone. Unsurprisingly, for most patients and most toxicities, the largest NTCP reductions were obtained when SUR and AGC are applied together.

**Figure 4.**
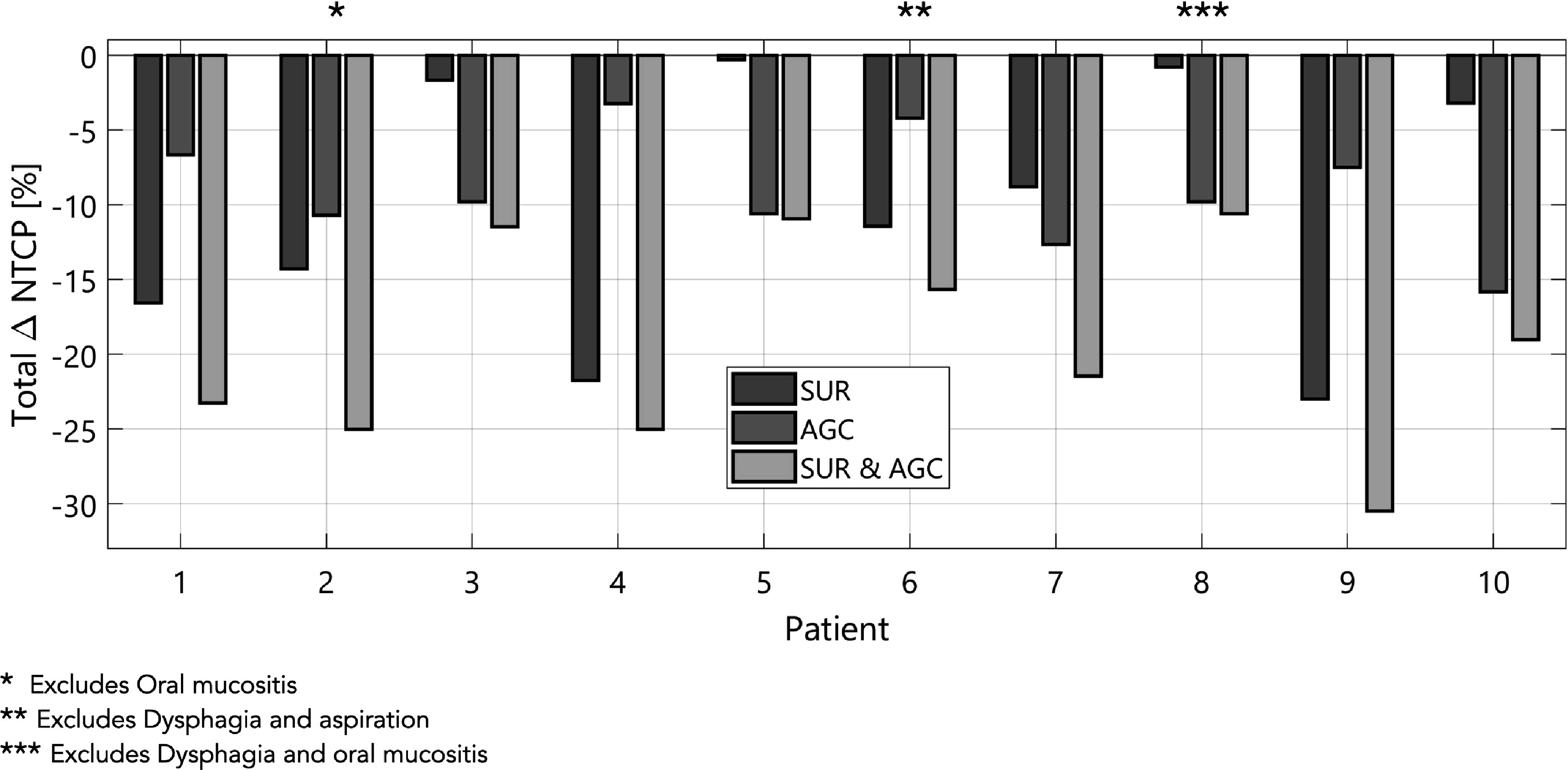
Total normal tissue complication probability (NTCP) differences for xerostomia, dysphagia, aspiration and oral mucositis for each patient when performing 3 mm setup uncertainty reduction (SUR), adaptation to geometrical changes (AGC) as well as SUR and AGC combined.

Figure 4 presents the patient-specific total NTCP reductions for the four main toxicities considered in this work that are derived from different anatomical structures: xerostomia, dysphagia, oral mucositis and aspiration. When SUR and AGC are performed together, reductions in the probability of developing any of these four toxicities ranged between 10.94 and 30.48 percentage points (pp) within our patient cohort. Focussing on NTCP reductions achieved by SUR and AGC separately, results in figure 4 indicates that AGC was more beneficial than SUR for half of the patient cohort, and SUR was more beneficial for the other half of the group. This contrasts with the results of figure 3, where toxicity-specific NTCP reductions averaged over the whole patient cohort were generally higher with SUR than AGC. A power analysis of the differences in total NTCP reductions induced by SUR, AGC and SUR&AGC combined is presented in the appendix.

**Figure 5.**
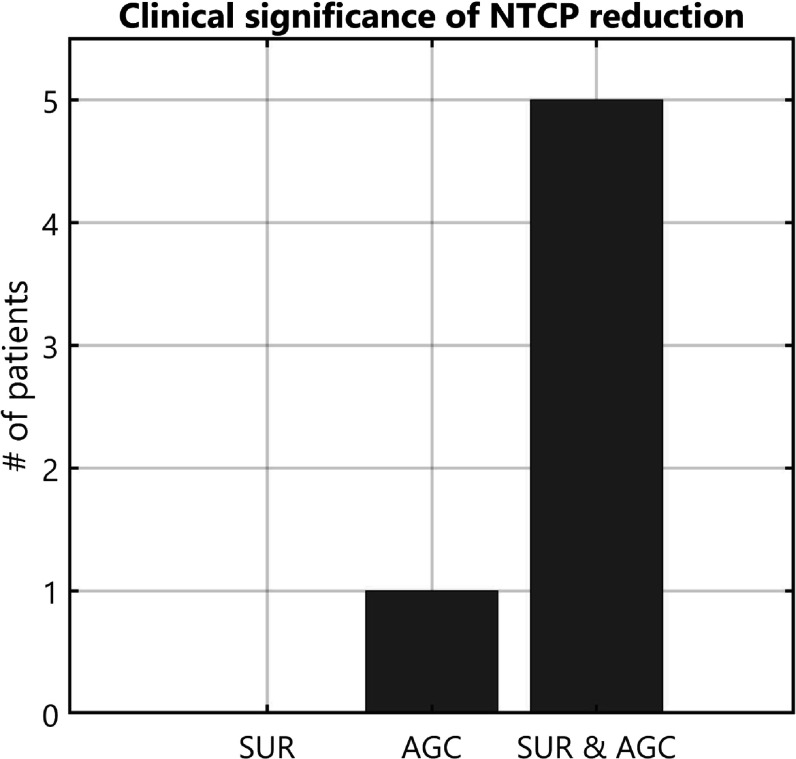
Number of patients with a clinically significant reduction in normal tissue complication probability (NTCP) when performing a 3 mm setup uncertainty reduction (SUR), adaptation to geometrical changes (AGC) with online adaptive proton therapy as well as SUR and AGC combined. Clinical significance is established using the decision tree presented in figure 2.

Finally, the proportion of patients identified to have a clinically significant NTCP reduction utilizing each mechanism according to the decision tree presented in figure 2 is reported in figure 5. Using SUR alone, no patient was identified to have a clinically meaningful NTCP reduction, compared to only one using AGC alone and five using AGC and SUR simultaneously.

## Discussion

In this study, a cohort of ten head and neck squamous cell carcinoma patients with daily scatter corrected CBCTs was used to retrospectively assess the reduction in NTCP induced by SUR and AGC in the context of online adaptive proton therapy. NTCP models for six different endpoints were calculated on the cumulative dose distributions from three different clinical scenarios to assess NTCP reductions associated with SUR, AGC and SUR and AGC combined. While previous studies have investigated the influence of the setup robustness setting on NTCP for head and neck IMPT (Van De Water *et al* 2016, Arts *et al* 2017, Wagenaar *et al* 2021), this work is the first to quantify the contributions of SUR and AGC on NTCP reductions achievable using online adaptive head and neck IMPT based on cumulative dose distributions.

The potential of OA to restore plan quality and limit the impact of anatomical changes on target coverage and the dose to the surrounding OARs has been highlighted in previous studies (Da Silva *et al* 2015, Jagt *et al* 2017, Botas *et al* 2018, Lalonde *et al* 2021, Bobić *et al* 2021, 2023). NTCP models implemented in this work showed that the OAR sparing achieved with online adaptative IMPT does translate into meaningful reductions in the risk of late toxicity when SUR and AGC are performed simultaneously. Among the six NTCP toxicities considered in this work, daily OA was shown to have the greatest impact on the risk of aspiration, dysphagia and xerostomia with mean reductions of 8.25, 5.42 and 5.12 pp respectively. While daily adaptation was used in this work as a proof of principle, it has been demonstrated that OAR sparing was comparable using daily and weekly OAs (Bobić *et al* 2021), suggesting that similar NTCP reductions as the one obtained in this study could be achieved using less frequent adaptations. Similarly, our results are expected to be representative of what would be achieved using CT-on-rails instead of scatter corrected CBCT as in-room imaging modality, since both were shown to provide clinically equivalent results in the context of head and neck online adaptive proton therapy (Nesteruk *et al* 2021, 2022).

Our analysis consisted of comparing NTCP associated with three scenarios utilizing different setup robustness settings (3 mm versus 0 mm) and adaptation schemes (fast daily OA versus no adaptation). As done in similar studies, our data analysis was focussed on NTCP differences between each scenario rather than absolute NTCP values (Van De Water *et al* 2016, Arts *et al* 2017, Wagenaar *et al* 2021), this to isolate the impact of selected mechanisms on NTCP while minimizing the impact of patient characteristics and institution-specific treatment planning protocols on our conclusions. When SUR alone is considered, our results are similar to what has been reported previously in terms of NTCP reductions achievable by decreasing the setup robustness setting. In our study, a mean reduction in NTCP using SUR of 0.93 pp mm^−1^ and 1.00 pp mm^−1^ was achieved for xerostomia and dysphagia respectively, compared to 1.0 pp mm^−1^ and 0.7 pp mm^−1^ in Wagenaar *et al* (2021). Differences between these studies are most likely explained by the fact that our analysis was based on cumulative dose distributions rather than simulated setup error scenarios.

Focussing on AGC alone, our results indicate that online adaptive proton therapy can induce NTCP reductions that go beyond what is achieved when applying SUR only. Indeed, total NTCP reductions per patient for xerostomia, dysphagia, aspiration and oral mucositis reported in figure 4 ranged between 3.25 and 15.84 pp when using AGC alone, and half of our cohort in fact benefited more from AGC than SUR alone based on the cumulative doses. This indicates that by simply readjusting the intensity of highly weighted beamlets daily, online adaptive proton therapy can limit the escalation of NTCP induced by the degradation of the plan quality. Interestingly, NTCP reductions associated with AGC were not uniform across all toxicities, as shown in figure 3. For instance, the risk for oral mucositis was below 3 pp for all patients using AGC, while for aspiration, reductions up to 10 pp were achieved. One possible explanation is the much larger inter-fractional motion amplitude of the larynx compared to organs in the oropharynx region (Gurney-Champion *et al* 2018), from which NTCP for oral mucositis is calculated. For SUR, more uniform mean NTCP reductions were achieved across the toxicities considered in this work, with the exception of solid swallowing problems, where both SUR and AGC had minor influence.

The clinical significance of the reductions in NTCP induced by SUR and AGC was assessed using a decision tree inspired by the Dutch model-based selection system (Tambas *et al* 2020). Based on this tree, half of our patient cohort (patients # 1, 2, 6, 9 and 10) was shown to have a clinically significant NTCP reduction when performing online adaptive proton therapy combining SUR and AGC. However, adapting to geometrical changes at a constant setup uncertainty robustness resulted in a clinically meaningful toxicity reduction for only one patient. Thus, it appears that despite its potential to address inter-fractional anatomical changes, OA is more likely to induce a clinically meaningful reduction in toxicity risks if it is also leveraged to reduce the setup uncertainty robustness setting. Likewise, reducing setup uncertainty margins by 3 mm was shown to induce no clinically meaningful toxicity reductions at the end of treatment if inter-fractional geometrical changes in the patient anatomy were not also addressed during treatment delivery. Considering that contrary to AGC, robustness reductions have the potential to compromise tumor control (Wagenaar *et al* 2021), it appears that SUR performed alone has little potential to improve the therapeutic index of head and neck IMPT.

The fact that clinically meaningful toxicity reductions were observed in five out of ten patients also illustrates the heterogeneous effect of online adaptive proton therapy on toxicity reductions across our patient cohort. This is even more apparent in figure 4, where half of the patient cohort had larger NTCP reductions with SUR than AGC alone, while the opposite was true for the other half. Although our work suggests that AGC and SUR should be implemented together to maximize clinical benefits, the variable effect of both mechanisms on each patient stresses out the challenge of identifying which kind of adaptation strategy is more suited for a given patient, if any. Clinical factors leading to meaningful NTCP reductions using OA could not be identified by this study due to our limited cohort size but neither the volumes of the CTVs or the amount of weight lost during treatment seemed to be indicative of the NTCP reduction’s amplitude achieved with both SUR and AGC. While challenging, identifying the subset of patients that would benefit the most from OA would be clinically relevant, as this could allow for a more efficient allocation of resources. In that regard, methods based on anatomical modeling (Gurney-Champion *et al* 2018, Zhang *et al* 2022), previously identified pre-treatment clinical factors (van Kranen *et al* 2013) or deep learning (Pakela *et al* 2021) should be investigated in the future.

This study had some limitations worth mentioning. First, uncertainties in image deformation, contour propagation and residual setup errors beyond treatment adaptation were neglected. NTCP reductions achieved in this work shall therefore be interpreted as the best-case scenario for AGC and might not be fully achievable in a clinical setting. However, it is also worth noting that several strategies have been suggested to mitigate the impact of these uncertainties in the context of online adaptive proton therapy: the integration of structure uncertainties in plan re-optimization (Nenoff *et al* 2022), the use of surface guidance to monitor patient positioning (Freislederer *et al* 2020) and prompt gamma imaging to assess setup errors (Hueso-González *et al* 2018). A second limitation is that our methodology assumed equivalent proton range uncertainty between CT and CBCT, which again represents a best-case scenario for scenarios involving AGC. In practice, proton therapy plans optimized on different imaging modalities require revisiting the margin recipe used to account for range uncertainty. We anticipate this simplification to only have a minor impact on this work, since proton ranges calculated on CBCT images corrected for scatter using our deep learning algorithm were shown to agree with CT within 0.66% (Lalonde *et al* 2020) and because range uncertainty has been shown to have a minimal effect (less than 0.5 pp/%) on NTCP in head and neck patients (Van De Water *et al* 2016, Wagenaar *et al* 2021). However, for cases where proton range prediction accuracy would significantly differ between offline and online imaging (for instance dual-energy CT for treatment planning (Bär *et al* 2017) and magnetic resonance for adaptation (Hoffmann *et al* 2020)), NTCP reductions reported in this work for AGC might not be achievable once the different range uncertainty regiments are properly accounted for.

Another limitation to consider is the fact that NTCP models were derived from photon patients. Blanchard *et al* (2016b) showed that photon-based NTCP models were valid in a cohort of head and neck proton therapy patients, suggesting that similar conclusions could have been drawn if we had used proton-specific NTCP models instead. However, other work has demonstrated that toxicity risk was influenced by voxel-level dose distributions which, for similar dose-to-organ, can be considerably different between photons and protons (Monti *et al* 2017). An evaluation of the benefits of online adaptative strategies using proton specific NTCP models could therefore be insightful. Finally, results presented in this study were focussed on NTCP values, as those are the metrics guiding modern model-based selection tools. The impact of adaptation on tumor control probability (TCP) was not addressed since the primary objective of OA is to restore target coverage, which will always improve TCP. Moreover, NTCP reductions associated with SUR and AGC could in theory be leveraged to perform dose-escalation for some patients, which would again increase TCP. Evaluating the potential of OA to increase the therapeutic window of head and neck IMPT through TCP and NTCP optimization was out of the scope of this work but warrants future investigation.

In conclusion, this study highlighted for the first time the impact of online adaptive proton therapy on toxicity risks. Combining effects from a reduction of the setup uncertainty setting by 3 mm and the AGC, online adaptive proton therapy based on a limited spot-intensity re-optimization workflow was shown to allow clinically meaningful toxicity reductions in 50% of our patient cohort, with total NTCP reductions up to 30.48 pp for the four main toxicities considered in this work.

## Figures and Tables

**Figure A.1.**
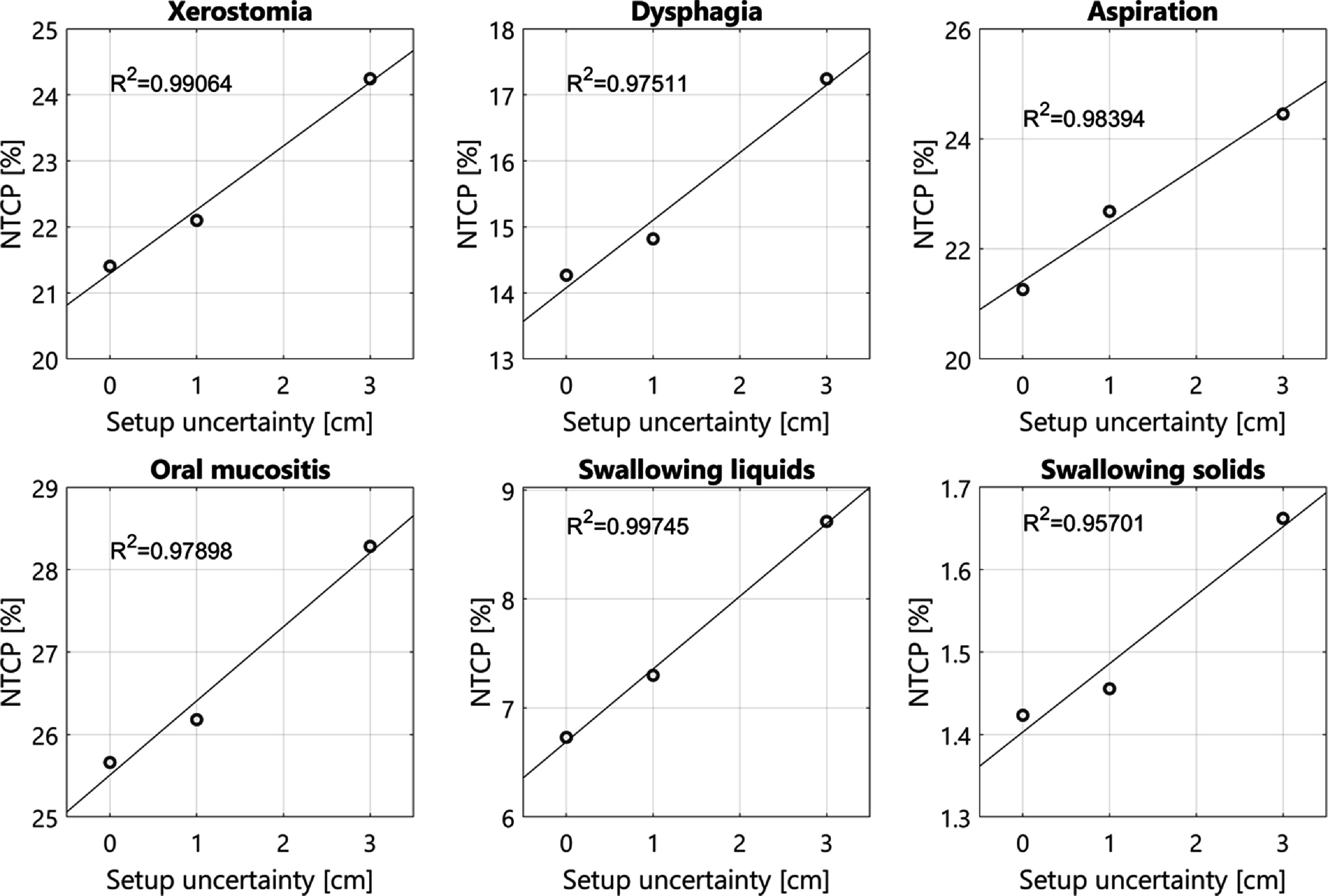
Mean normal tissue complication probability (NTCP) as a function of the in the setup uncertainty setting used during robust optimization.

**Figure A.2.**
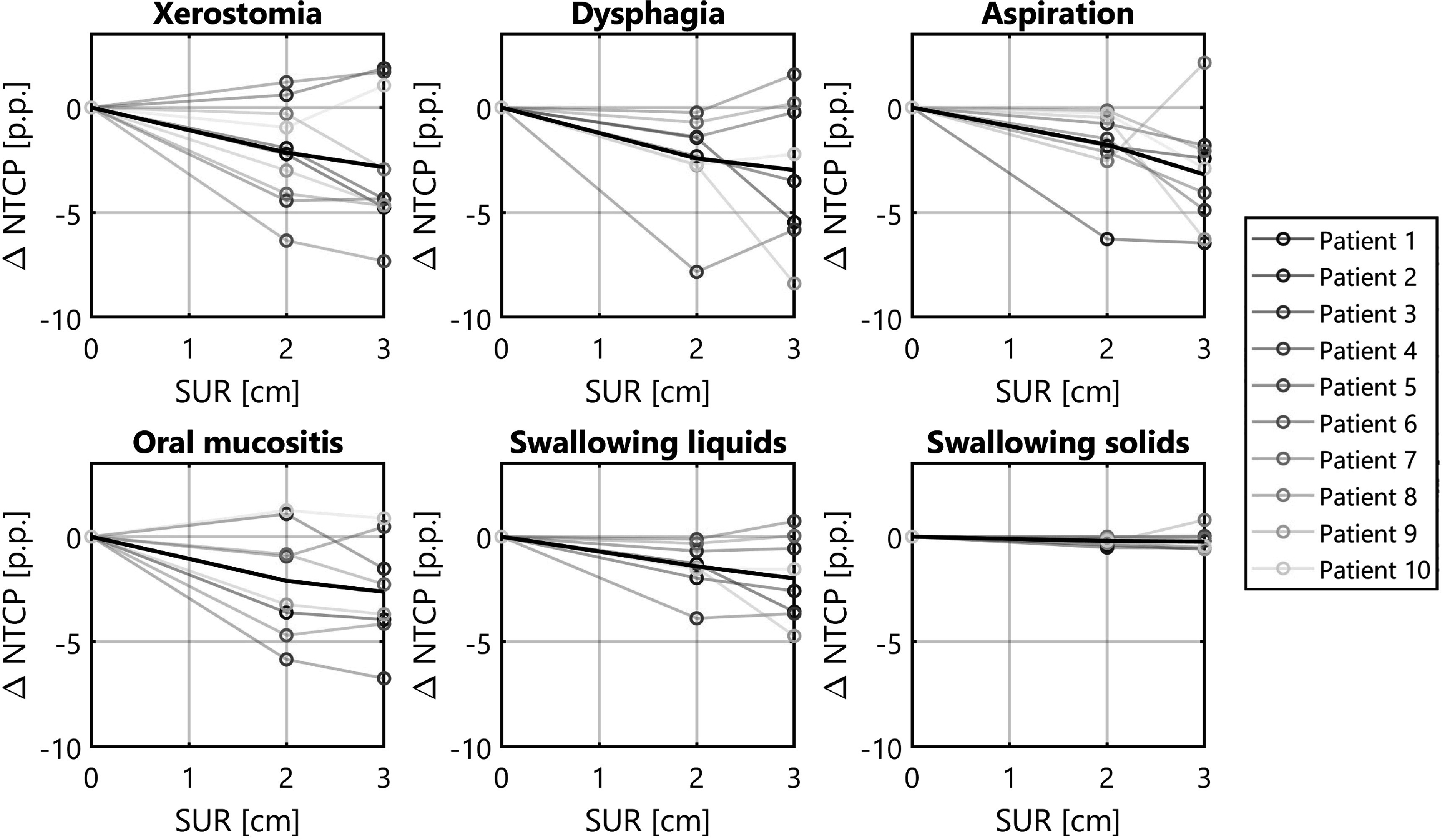
Normal tissue complication probability (NTCP) differences as a function of the reduction in the setup uncertainty setting used during robust optimization. The black lines report the mean of the cohort.

**Figure A.3.**
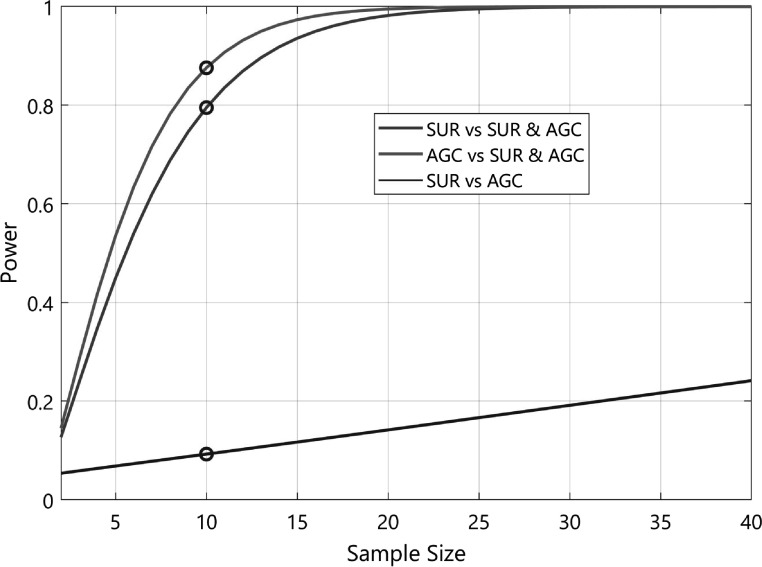
Power of the two-sample t-test comparing total NTCP reductions induced by each mechanism as a function of the sample size.

## Data Availability

The data cannot be made publicly available upon publication because they are not available in a format that is sufficiently accessible or reusable by other researchers. The data that support the findings of this study are available upon reasonable request from the authors.

## Appendix

### Linearity of NTCP with setup uncertainty setting

In this work, the impact of a 3 mm SUR was evaluated by computing differences in NTCP for cumulative doses obtained with plans optimized using a 3 mm setup uncertainty robustness and plans optimized with no explicit setup robustness (or, equivalently, a 0 mm setup uncertainty). Previous work has demonstrated that NTCP for common head and neck toxicities is linearly related to the setup uncertainty setting within 1 and 5 mm, suggesting that any 3 mm SUR within that range would have an equivalent impact on NTCP. To evaluate whether this relationship is valid down to 0 mm, we simulated an additional scenario considering robustly optimized plan using a 1 mm setup uncertainty for all ten patients. Cumulative doses obtained with that scenario were compared to scenarios A and B to assess the impact on NTCP of SUR of 2 and 3 mm. Results presented in figure A.1 demonstrate a linear behavior of the mean NTCP differences between all three levels of setup uncertainty (0, 1 and 3 mm). Similarly, figure A.2 shows the evolution of NTCP reductions achieved with a SUR of 0 mm (which by definition implies no NTCP difference), 2 and 3 mm on a patient per patient basis. Once again, a linear behavior is observed in terms of mean NTCP reduction for most toxicities.

### Power analysis

To assess the statistical significance of the differences in NTCP reductions induced by each mechanism considered in this work, a power analysis was performed using a two-sample t-test based on the total NTCP reductions associated with xerostomia, dysphagia, aspiration and oral mucositis considering a significance level of 0.05. Results presented in figure A.3 show a power larger or equal to 79% for SUR versus SUR and AGC as well as AGC versus SUR and AGC with the current cohort size.
